# Improving Reproducibility of Eddy-Current-Based Coating Thickness Estimation with Printed Circuit Board-Based Differential Coils †

**DOI:** 10.3390/ma19153201

**Published:** 2026-07-27

**Authors:** Martin Koll, Bernhard Salcher, Markus Peer, Daniel Wöckinger, Gerd Bramerdorfer, Stefan Schuster, Stefan Scheiblhofer, Norbert Gstöttenbauer, Johann Reisinger

**Affiliations:** 1 Institute of Electric Machines and Power Electronics, Johannes Kepler University Linz, Altenbergerstraße 69, 4040 Linz, Austria; 2 voestalpine Stahl GmbH, Voestalpine-Straße 3, 4020 Linz, Austria

**Keywords:** analytical modeling, coating thickness, eddy current testing, model-based estimation, multi-layer pancake coil

## Abstract

The accurate determination of the thickness of the metallic coating on steel substrates is essential in industrial quality control. Eddy current testing offers a non-destructive solution by evaluating the impedance or mutual impedance of one or multiple coils. Analytical models exist in the literature for selected sensor configurations. Building on these models, a model-based estimation approach can be applied to derive an estimate for coating thickness and other relevant material and geometry parameters. Conventional setups typically employ wire-wound coils. However, manufacturing tolerances introduce discrepancies between nominal and actual coil geometries, which lead to deviations in the coating thickness estimate. A printed circuit board (PCB)-based coil with lithographically defined geometry achieves substantially tighter fabrication tolerances and higher repeatability than wire-wound coils. In this work, an analytical mutual impedance model for a differential multi-layer PCB pancake coil is derived and validated against established models in the literature with respect to forward modeling accuracy and model-based parameter estimation performance. Furthermore, experimental measurements with two-layer and eight-layer PCB differential coil systems produce parameter estimates with significantly better reproducibility than wire-wound coils. The experimental results show that the two-layer PCB coil achieves close agreement between the absolute estimated parameters and the reference values without requiring additional calibration, while maintaining high sensitivity to coating thickness.

## 1. Introduction

**Figure 1:**
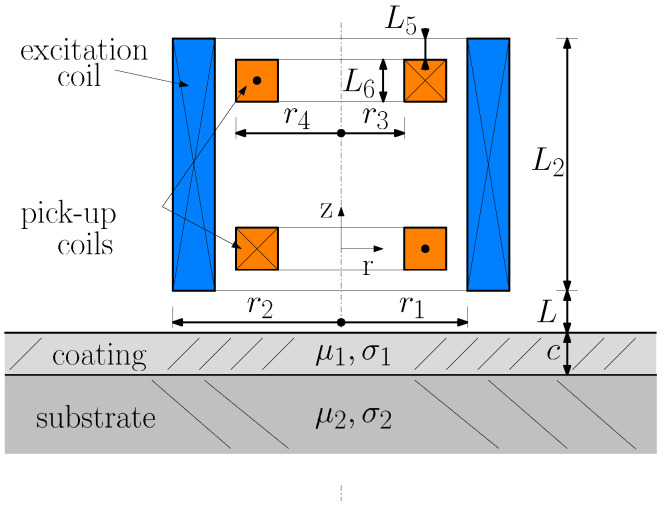
Illustration of wire-wound differential coil system: coarse model approximation (adapted from [14]).

Eddy current testing (ECT) is a widely used non-destructive testing technique for assessing electrically conductive materials. Both multi-frequency approaches and pulsed eddy current (PEC) testing can be used for material inspection [1,2]. These approaches are particularly effective for detecting surface and near-surface defects and for characterizing key material properties such as conductivity, permeability, and coating thickness [3]. In [4], recent developments in ECT approaches for planar structures are reported. An important industrial application is the inspection of metal sheets with thin conductive coatings on conductive ferromagnetic steel substrates. In this work, a differential coil sensor concept with increased sensitivity to material parameters was used. A schematic of the ECT configuration consisting of an excitation coil and two anti-serially coupled pick-up coils next to coated sheet metal is shown in Figure 1. For this configuration, analytical eddy current (EC) mutual impedance models are available in the literature [5] and, therefore, enable a model-based parameter estimation approach [6,7,8,9,10].

However, a drawback of classical wire-wound ECT coils is their vulnerability to manufacturing tolerances [11,12], e.g., coil bobbins, winding dimensions, and relative coil positioning, which introduce deviations from the nominal geometry. Furthermore, the large number of geometric parameters of the differential coil setup and their strong correlations in the mutual impedance measurement make accurate calibration challenging.

When a model-based estimator assumes nominal coil geometry, any discrepancy between the actual and modeled geometry can cause biased parameter estimates. This motivated the usage of printed circuit board (PCB)-based differential coils with tighter, lithographically defined tolerances to improve the reproducibility of coil fabrication and the accuracy of subsequent parameter estimation.

In [13], the influences of the coil geometry and manufacturing tolerances on the inductance variation of planar spiral multi-layer PCB coils were studied. This study shows that even small manufacturing tolerances can lead to changes in the coil’s inductance, especially in PCB coils with multi-layer structures. However, in practical ECT, these effects are not limited to the coil characteristics themselves. Instead, they propagate through the entire measurement and directly affect the accuracy of the inverse-model-based parameter estimation approach and therefore lead to estimation bias.

For selected PCB coil setups, analytical EC models have been developed and investigated. For instance, coplanar rectangular spiral coils have been modeled for the characterization of conductive plates, demonstrating practical relevance [15]. Differential coil concepts have further enhanced sensitivity by exploiting subtraction-based measurement principles, which improve robustness against common-mode disturbances and increase sensitivity to material property variations [16]. Recent reviews on ECT probe design highlight the growing interest in PCB-based sensor architectures, which is due to their precisely manufactured geometry and compatibility with scalable manufacturing processes [17]. Beyond sensor design aspects, reproducibility also plays a critical role in reproducible electromagnetic material characterization. In this context, PCB-based implementations have been shown to improve measurement consistency in magnetic material characterization compared to conventional wound coil systems [18]. Despite previous work, a systematic quantification of how PCB implementation choices in differential multi-layer pancake coil probes affect both reproducibility and estimation bias in model-based inversion of metallic-coated ferromagnetic steel sheets is still lacking. This work addresses this aspect through an experimental comparison of multiple coil designs and realizations. The main contributions of this work are summarized as follows:

- Generalization of an analytical EC model for a PCB-based differential multi-layer eddy current pancake coil.
- Development and experimental comparison of wire-wound and PCB-based differential coil probes for ECT coating thickness estimation.
- Quantitative assessment of the reproducibility of PCB coil manufacturing compared to conventional wire-wound coil systems.
- Investigation of the propagation of coil geometry deviations through a model-based inversion framework and their impact on coating thickness estimation accuracy.
- Quantification of estimation bias and variability induced by manufacturing tolerances in differential coil EC sensors.
- Demonstration of the applicability of PCB-based differential coil systems for inspection of coating on ferromagnetic steel substrates.

## 2. Methodology

Studying the reproducibility of model-based coating thickness estimation with PCB coils requires an analytical EC model, a validation of the model, and measurement comparisons with traditional wire-wound coils. Building on the analytical EC model of Dodd and Deeds [5], an exact analytical model tailored to a differential multi-layer PCB pancake coil geometry was derived. It was validated via finite element simulations performed using FEMM [19]. Then, the derived PCB model was benchmarked against the classical coarse model approximation of Dodd and Deeds [5]. In the coarse model, only the outer contour of the PCB coil cross-section is considered, as shown in Figure 1, whereas the fine PCB model explicitly resolves each individual winding in every PCB layer. Additionally, the suitability of the coarse model approximation concerning a model-based estimation approach with PCB coils for determining material parameters is studied. To validate the theoretical assumptions regarding coating thickness estimation reproducibility, nine realizations of 2- and 8-layer PCB-based differential coils were designed and fabricated.

The 2-layer PCB design was expected to be highly reproducible because it uses a single dielectric core and maintains tight tolerances between the two copper layers. Its drawback is reduced sensitivity to material parameters due to the small number of turns. Therefore, 8-layer PCBs were also investigated, as they keep manufacturing costs low while offering higher sensitivity but very likely are less reproducible during manufacturing and therefore cause a higher variance in parameter estimates.

The PCB-based measurement results were compared with measurements obtained from two wire-wound differential coils. Measurements were conducted on the test bench described in [20] using electrical steel sheet samples with oven-cured silver-ink coatings with thicknesses of 8 μm and 21 μm for fixed liftoff conditions. For the measurements, the excitation coils were driven with a sinusoidal current, and the induced differential voltage was measured in the pick-up coils.

The coating thickness was subsequently estimated using a model-based approach based on the analytical EC model. Finally, the reproducibility was evaluated by analyzing the deviations of the estimated coating thickness from the respective mean value obtained for each manufactured coil setup. In this case, the mean value was used as a reference in order to suppress additional systematic influences that were not directly related to the coil manufacturing reproducibility. In particular, effects such as variations in the skin and proximity effects within the excitation winding, parasitic effects, as well as other coil-specific setup differences, were thereby reduced. Consequently, the investigation in this paper focuses primarily on the reproducibility of the coil fabrication and the associated model-based parameter estimation.

In addition, microsection images of the manufactured PCB coils were acquired to evaluate how accurately the fabricated geometries correspond to the manufacturer’s specifications.

In addition to the deviations relative to the estimated mean values, the estimated parameters were also compared to the corresponding known reference coating thickness values in order to assess the absolute estimation accuracy. This additionally allows distinguishing between high reproducibility and systematic estimation bias and identifies which coil configurations enable accurate absolute parameter estimation without additional calibration.

## 3. Analytical PCB Coil EC Model

**Figure 2:**
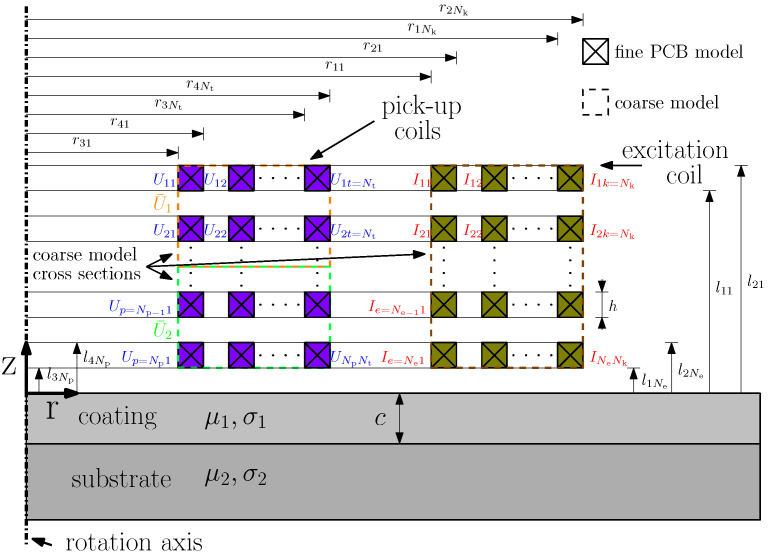
PCB differential coil setup with excitation and pick-up system with $N_{p}=N_{e}$ layers. Shown are the coarse (outline-only) and refined (winding-resolved) cross-sections of the considered analytic models (adapted from [21]).

This section presents an improved analytical EC model of a differential PCB coil based on modifications of [5]. An illustration of the layered PCB configuration next to a coated steel sheet is shown in Figure 2. All the excitation windings are connected in series. Additionally, the upper and lower pick-up windings are each connected in series but wound in opposite directions. The resulting differential voltage between the two pick-up coils is evaluated. This differential connection leads to increased sensitivity to sheet metal parameters, as the induced voltage of the air-core coil is compensated. Both pick-up coils each occupy half of the total number of PCB layers. The complex valued voltage $U_{pt}$ induced in a single pick-up winding can be described as a function of the currents $I_{ek}$ in all excitation windings by [21]

(1) $$\begin{array}{c} U_{pt}=\sum_{e=1}^{N_{e}}\sum_{k=1}^{N_{k}}\frac{j\omega \mu_{0}\pi I_{ek}}{h(r_{4t}-r_{3t})}\int_{0}^{\infty }\frac{1}{\alpha^{6}}J\left(r_{2k},r_{1k}\right)J\left(r_{4t},r_{3t}\right)\Phi_{p}\;d\alpha . \end{array}$$

Three case distinctions in $\Phi_{p}\left(R\left(\alpha \right)\right)$ for p>e, p<e and p=e are necessary. The index *p* describes the selected layer of the pick-up winding, and *e* is the layer of the excitation winding under consideration in (1). The index *t* describes the radial position of the pick-up coil winding, and *k* is the radial position of the excitation coil winding. $N_{p}$ is the number of pick-up coil layers, and $N_{e}$ represents the number of excitation coil layers. It holds that: $N_{p}=N_{e}$. $N_{t}$ represents the number of adjacent turns per layer in the pick-up coils, and $N_{k}$ is the number of adjacent turns per layer in the excitation coil.

Case 1: $\Phi_{p}\left(R\left(\alpha \right)\right)$ for p<e:
  (2) $$\begin{array}{cc} \Phi_{p} & =\left(e^{-\alpha l_{3p}}-e^{-\alpha l_{4p}}\right)\\ \; & \;\times \left(e^{\alpha l_{2e}}-e^{\alpha l_{1e}}-R\left(\alpha \right)\left(e^{-\alpha l_{2e}}-e^{-\alpha l_{1e}}\right)\right), \end{array}$$
Case 2: $\Phi_{p}\left(R\left(\alpha \right)\right)$ for p>e:
  (3) $$\begin{array}{cc} \Phi_{p} & =\left(e^{-\alpha l_{1e}}-e^{-\alpha l_{2e}}\right)\\ \; & \;\times \left(e^{\alpha l_{4p}}-e^{\alpha l_{3p}}-R\left(\alpha \right)\left(e^{-\alpha l_{4p}}-e^{-\alpha l_{3p}}\right)\right), \end{array}$$
Case 3: $\Phi_{p}\left(R\left(\alpha \right)\right)$ for p=e:
  (4) $$\begin{array}{cc} \Phi_{p} & =2\alpha \left(l_{4p}-l_{3p}\right)-e^{\alpha (l_{4p}-l_{2e})}+e^{\alpha (l_{3p}-l_{2e})}+e^{-\alpha (l_{4p}-l_{1e})}\\ & \;-e^{-\alpha (l_{3p}-l_{1e})}+R\left(\alpha \right)\left(e^{-\alpha l_{1e}}-e^{-\alpha l_{2e}}\right)\\ & \;\times \left(e^{-\alpha l_{3p}}-e^{-\alpha l_{4p}}\right). \end{array}$$

Additionally, the relationships

(5) $$\beta_{i}=\frac{1}{\mu_{i}}\sqrt{\alpha +j\omega \mu_{0}\mu_{i}\sigma_{i}},$$

(6) $$\frac{1}{\alpha^{2}}\int_{x=\alpha r_{1}}^{\alpha r_{2}}xJ_{1}\left(x\right)\;dx=\frac{1}{\alpha^{2}}J\left(r_{2},r_{1}\right),$$

where $J_{1}\left(x\right)$ is the Bessel function of the first kind and first order and the sheet factor R(α) for a two-layered metal sheet

(7) $$R\left(\alpha \right)=\frac{\left(\alpha +\beta_{1}\right)\left(\beta_{1}-\beta_{2}\right)+\left(\alpha -\beta_{1}\right)\left(\beta_{1}+\beta_{2}\right)e^{2\alpha_{1}c}}{\left(\alpha -\beta_{1}\right)\left(\beta_{1}-\beta_{2}\right)+\left(\alpha +\beta_{1}\right)\left(\beta_{1}+\beta_{2}\right)e^{2\alpha_{1}c}}$$

where $\alpha_{i}=\mu_{i}\beta_{i}$, are required. The sheet factor *R* assumes linear, isotropic, and homogeneous material and can be extended to a multi-layer structure, as shown in [22]. For selected conductivity and permeability profiles, solutions are given in [7,23]. ω=2πf is the angular frequency, and *f* denotes the excitation frequency. The index *i* describes the different layers in the sheet metal. Furthermore, the layers’ relative permeability and conductivity are defined as $\mu_{i}$ and $\sigma_{i}$, respectively. If the coating is assumed to be diamagnetic, in contrast to a ferromagnetic substrate material, its permeability can be assumed to be $\mu_{1}=1$. $\mu_{0}$ is the permeability in a vacuum. The required lengths and radii in (1)–(6) are shown in Figure 2. The coating thickness is defined by *c*, and α is the integration variable. It is assumed that the copper thickness *h* is the same in all PCB layers. Then, the voltage difference between the pick-up coils can be calculated by $U={\bar{U}}_{1}-{\bar{U}}_{2}$ with

(8) $$\begin{array}{c} {\bar{U}}_{1}=\sum_{p=1}^{\frac{N_{p}}{2}}\sum_{t=1}^{N_{t}}U_{pt},\;\mathrm{and}\;{\bar{U}}_{2}=\sum_{p=\frac{N_{p}}{2}+1}^{N_{p}}\sum_{t=1}^{N_{t}}U_{pt}. \end{array}$$

Due to the serial connection of all excitation coil conductors, the excitation currents are assumed to be $I_{11}=I_{21}=\cdots =I_{N_{e}N_{k}}\equiv I$. In the coarse model approximation, the parameters are given by $r_{1}=r_{11}$, $r_{2}=r_{2N_{k}}$, $r_{3}=r_{31}$, $r_{4}=r_{4N_{t}}$, $l_{1}=l_{1N_{e}}$, $l_{2}=l_{21}$, $L_{5}=0$, and $L_{6}=\left(l_{21}-l_{1N_{e}}\right)/2$.

The presented model resolves each individual turn of the coil and is referred to as the winding-resolved (fine) model in Figure 2. In contrast, an alternative modeling approach uses only the coil outlines as cross-sectional areas for the excitation and pick-up coils, which is referred to as the outline-only (coarse) model.

For model verification, an unrealistic worst-case 2-layer PCB coil with a large deviation between the fine PCB model cross-sections and coarse model cross-sections is considered, with $I_{11}=I_{12}=1\;A$, $r_{3}=25\;\mathrm{mm}$, $r_{4}=25.5\;\mathrm{mm}$, $r_{1}=30.5\;\mathrm{mm}$, $r_{2}=31\;\mathrm{mm}$, L=1mm, $L_{2}=10\;\mathrm{mm}$, $L_{5}=0$, $L_{6}=h/2$, h=2mm, and the material parameters are $\mu_{1}=1$, $\sigma_{1}=15\;\mathrm{MS}/m$, $\mu_{2}=1000$, and $\sigma_{2}=5\;\mathrm{MS}/m$. A frequency sweep with ten linearly spaced frequencies from $f_{\min}=10\;\mathrm{kHz}$ to $f_{\max}=200\;\mathrm{kHz}$ is used.

**Figure 3:**
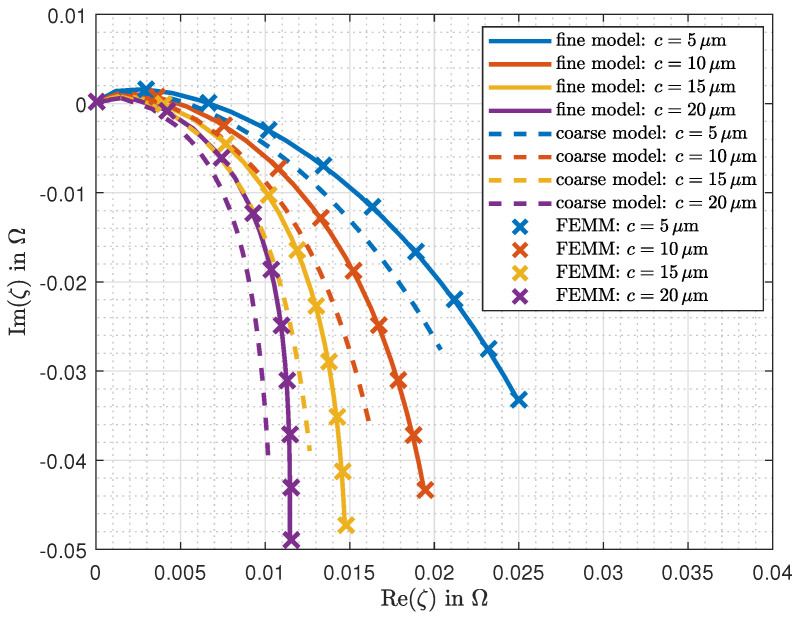
Comparison of a 2-layer PCB’s mutual impedance using finite element analysis, a fine analytical model, and the classical coarse model approximation by Dodd and Deeds [5] (adapted from [21]).

The mutual impedance is then calculated as ζ=U/I, which results for four different coating thicknesses in Figure 3. These model evaluations confirm the fine analytical model by finite element simulations in FEMM and show deviations from the coarse model approximation.

## 4. Model Investigations

An important point for model-based parameter estimation is the applicability of the coarse analytical model when a PCB coil is used for measurements. The choice between the coarse model and the fine model is critical when runtime matters, because computational cost grows with the number of layers and adjacent number of turns. The main advantage of the coarse model is the significantly lower computation time and the simpler implementation. The required computation time of the fine model scales with $O(N_{e}\;\cdot \;N_{k}\;\cdot \;N_{p}\;\cdot \;N_{t})$. Alternative analytical modeling approaches, such as truncated-region eigenfunction expansion methods [24], could result in a solution with significantly reduced computational time. A more computationally efficient coarse EC model for differential coils compared to [5] can be found in [25].

In this section, different PCB designs with varying numbers of layers are analyzed by the coarse and fine analytical EC models. Therefore, industrial PCB stack-ups from the company Eurocircuits were used. To achieve comparable mutual impedance results for the different kinds of PCB coils, $N_{t}=N_{k}=4$ adjacent turns per layer in the excitation coil and in the pick-up coils are used to create similar coil geometries. Each pick-up coil consists of $N_{p}/2$ layers. The total heights of the different PCB coils are around 1.5mm and are therefore similar. For the stack-ups of the 2- and 8-layer PCB coils, the data from Table 1 were used. Furthermore, for the investigated 12-layer PCB, a similar stack-up from Eurocircuits was employed.

**Figure 4:**
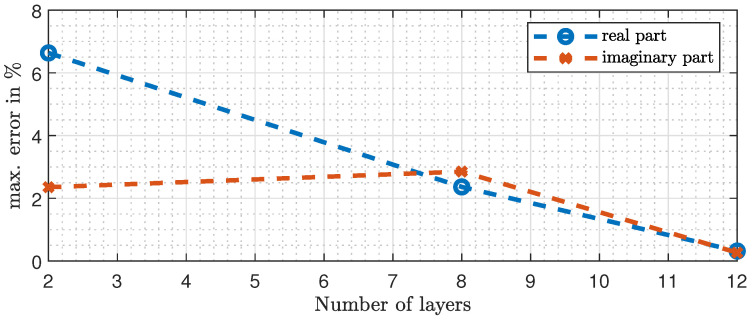
The results for the maximum error between the fine and the coarse analytical model in the real and imaginary parts of the mutual impedance for PCBs with different numbers of layers.

The maximum error in the mutual impedance between the fine PCB model and the coarse model is defined as the quality metric. Therefore, the same material parameters used for the investigation, illustrated in Figure 3 and described in Section 3, are employed. The resulting error plot is shown in Figure 4. It shows the expected trend. With increasing layer number, the maximum mutual impedance error decreases. This can be explained by improved coarse model coverage as the number of layers increases, while the overall PCB thickness remains constant. Nevertheless, the applicability of the coarse model to a given PCB design must be checked case-by-case. The error strongly depends on the selected stack-up, geometry, number of turns, and the specimen under test.

Typically, one would now be interested in determining material parameters using model-based estimation methods based on measured mutual impedance curves.

### Model-Based Estimation Approach

To assess the impact of the usage of the coarse analytical model instead of the fine PCB coil model on parameter estimation, the model-based inverse problem is defined as a least-squares fit over all excitation frequencies:

(9) $$J=\sum_{f=f_{\min}}^{f_{\max}}{\left(\zeta_{\mathrm{coarse}\text{-}\mathrm{model}}\left(f,\theta \right)-\zeta_{\mathrm{fine}\text{-}\mathrm{model}}\left(f,\theta \right)\right)}^{2}.$$

The estimator minimizes the deviation between the coarse-model prediction and the reference response obtained by the fine PCB model or measurements for real ECT application. By minimizing this cost function, estimates $\hat{\theta }$ for the unknown parameters can be given by

(10) $$\hat{\theta }=\mathrm{argmin}\left(J(f,\theta )\right).$$

**Figure 5:**
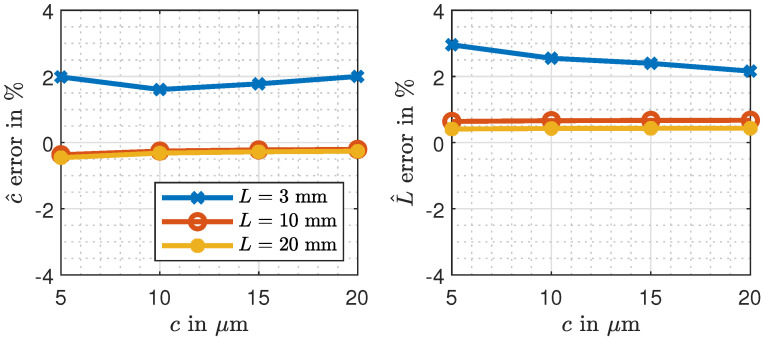
The relative error in the coating thickness $\hat{c}$ and liftoff estimate $\hat{L}$ caused by using the coarse model approximation for the 2-layer PCB.

Thereby, $\hat{\theta }$ collects all the unknown parameters, e.g., coating thickness, liftoff, or permeability. Minimization is performed using a Levenberg–Marquardt algorithm [26]. In this analysis, the worst case of the 2-layer coil, as shown in Figure 4, is analyzed to investigate the impact of model mismatch. Thereby, the focus is coating thickness estimation. Therefore, a two-parameter estimator with θ=(c,L) is implemented. In the past, it was determined that the coating conductivity $\sigma_{1}$ must be accurately known to estimate the coating thickness *c* [6,20,27]. In this preliminary investigation, it is assumed that the electromagnetic substrate parameters are known in advance. The relative errors of the estimated coating thickness and liftoff, as functions of the true coating thickness, are shown in Figure 5.

It can be seen that the relative error decreases with increasing liftoff. Moreover, the maximum error remains at a very low level for all investigated coating thicknesses and is acceptable for most applications. In industrial applications, modeling accuracy is often more strongly affected by simplifying assumptions, such as an idealized planar sheet geometry or linear, homogeneous, and isotropic material properties [28]. The usage of the classical coarse model establishes a practical trade-off between model fidelity and computational efficiency for PCB-based differential ECT sensors.

## 5. PCB Coil Design

The use of PCB coils was motivated by observed deviations in parameter estimates across different wire-wound coils. These deviations arise from manufacturing tolerances inherent in the winding processes. PCB fabrication, in contrast, offers lithographically defined tolerances that are substantially smaller, thereby improving repeatability and reducing estimator bias and variation.

The analytical PCB coil model assumes concentric turns. Instead, spiral turns were implemented in the manufactured PCB coils. Due to the small conductor trace widths, the resulting deviation from the concentric-turns assumption was minor and was neglected for the considered designs. Crucially, turn symmetry across all layers was maintained so that residual asymmetries introduced by spiral routing cancelled due to the differential working principle.

For this investigation, 2- and 8-layer PCB coils were designed and fabricated. The designs were selected via numerical model simulation to maximize the sensitivity of the measured differential voltage with respect to coating thickness variations, subject to a constraint on the maximum outer coil radius and the stack-up in Table 1. The outer coil diameter was chosen based on the coating thickness on the samples being assumed to be sufficiently homogeneous over the investigated area, such that higher spatial resolution was not required. This assumption is consistent with typical industrial ECT coating thickness measurements, where spatial averaging is acceptable, and local defect resolution, e.g., crack detection [29,30], is not the primary objective and should be avoided. The geometry parameters of the PCB coils are summarized in Table 2 and defined as follows: excitation coil inner radius $r_{\mathrm{EC}}$, inter-turn spacing *s*, number of excitation turns $N_{1}$, excitation coil trace width $t_{\mathrm{EC}}$, pick-up coil inner radius $r_{\mathrm{MC}}$, number of turns $N_{2}$ per pick-up coil, and pick-up coil trace width $t_{\mathrm{MC}}$. For the approximation using the coarse model, the geometric relations are given by $r_{1}=r_{\mathrm{EC}}$, $r_{2}=r_{\mathrm{EC}}+\frac{N_{1}}{2}\;t_{\mathrm{EC}}+\left(\frac{N_{1}}{2}-1\right)$, $r_{3}=r_{\mathrm{MC}}$, and $r_{4}=r_{\mathrm{EC}}-s$. Furthermore, the heights are defined as $L_{5}=0$, $L_{6}$ is the height of one pick-up coil, and $L_{2}$ is the total height of the excitation coil.

**Figure 6:**
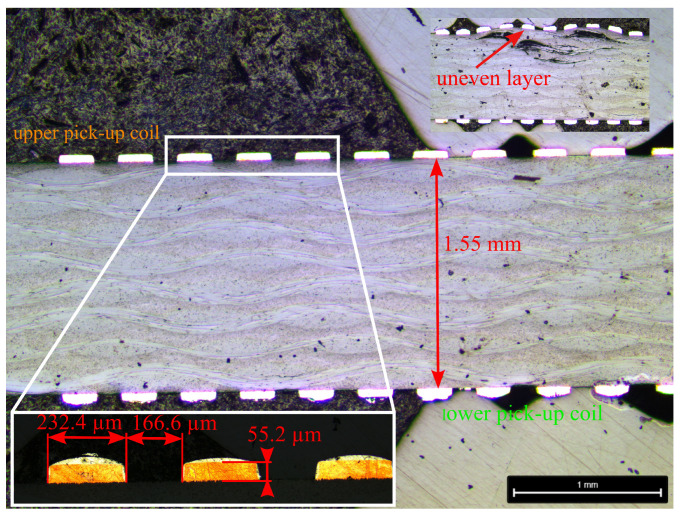
Microsection images of the 2-layer PCB pick-up coil configuration. An optimally manufactured coil with nominal dimensions indicated and a PCB with locally non-uniform core thickness resulting in an uneven conductor plane.

Fabrication was performed with standard tolerances. This choice kept costs low and enabled assessment of how typical production tolerances impact coating thickness estimation, reflecting the most common ordering practice for PCB coils. The employed PCB stack-ups are summarized in Table 1. To examine the internal structure of the PCB coils, metallographic cross-sections were prepared. Figure 6 shows microsection images of the 2-layer PCB pick-up coil and some details of the copper trace widths manufactured. A deviation from a nominal build-up is visible in the top-right corner. There, the core thickness is locally non-uniform, leading to a slightly uneven conductor plane. Furthermore, the copper thickness is noticeably larger than specified in the stack-up, while the final trace width is narrower than specified, resulting in a larger inter-trace spacing. The overall core thickness is in agreement with the stack-up specification.

**Figure 7:**
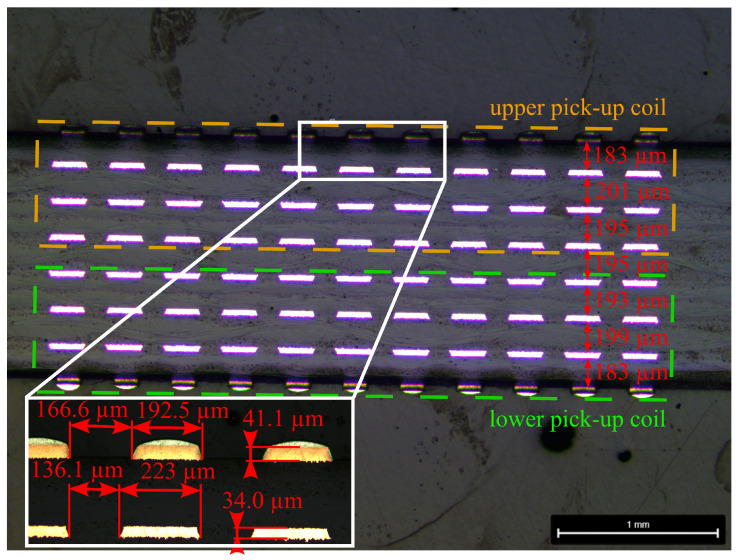
Microsection images of 8-layer PCB pick-up coil configuration.

Figure 7 shows a microsection and details of the 8-layer PCB pick-up coil stack-up. A slight lateral misalignment between layers is visible, and the trace widths exhibit small variations. Furthermore, the copper in the top and bottom layers is noticeably thicker, and the corresponding trace widths are smaller compared to those of the inner layers.

It should be noted that the layer stack-up may vary across fabrication lots. Thus, the reproducibility of the observed stack-up in a subsequent order cannot be guaranteed.

## 6. Wire-Wound Coil

To demonstrate the improvement in measurement reproducibility achieved with PCB coils, a comparison with wire-wound coils is shown. Because manual winding is labor-intensive, the sample size was limited to two coils. Prior investigations indicate that the pick-up coil height and its vertical symmetry with respect to the excitation coil are the main contributors to variations in the mutual impedance compared to the nominal value. For this purpose, impedance data were generated using the analytical model with vertically displaced pick-up coils, and the resulting influence on the model-based layer thickness estimation was investigated assuming nominal geometrical dimensions. For example, a vertical asymmetry of 100 μm between the measurement coils of the used coil setup shown in Table 3 resulted in a 11% deviation of the estimated coating thickness from the true value. This vertical symmetry is also the most difficult factor to control during manual winding. In addition, wire-wound coils are subject to geometric tolerances in all other dimensions, which further increase the deviation of the estimated parameters from their nominal values. As shown in Figure 1, the pick-up coils should have equal height $L_{6}$ and recess $L_{5}$. During fabrication, care was taken to manufacture both coils as identically as possible. The wire lengths were matched and routed as twisted pairs.

## 7. Measurement Results

**Figure 8:**
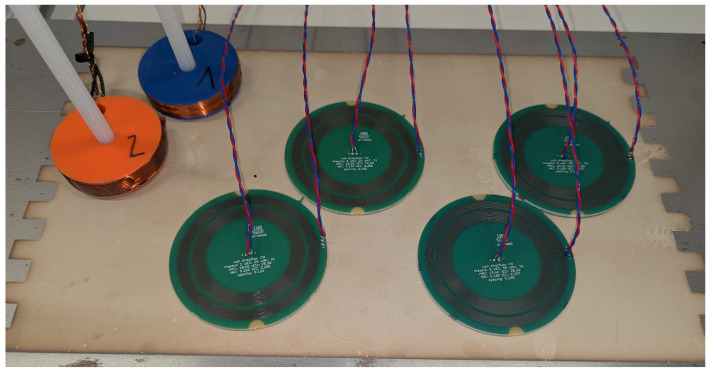
Photograph of two manufactured wire-wound coils and two selected coil realizations of 2-layer and 8-layer PCB designs placed on coated sheet sample.

The same measurement setup as in [20] was used. The electrical conductivity of the electrical steel sheet was $\sigma_{2}=3.054\;MS/m$, and its thickness was 0.5mm. The conductivity of the silver-ink coating was $\sigma_{1}=6.94\;MS/m$. This type of sample was chosen because it exhibits highly homogeneous material properties in both the coating and the substrate, as well as a well-defined and sharp interface between the two layers, thereby closely satisfying the assumptions of the analytical model. To ensure highly comparable results, a spacer was used to set a fixed air gap of L=3.34mm between the coil and the sheet specimens for the wire-wound coil and L=3mm for the PCB coils. All coils were rigidly fixed with weights in this position. The excitation current amplitude was actively controlled to I=0.1A for the wire-wound coils and to I=0.05A for the PCB coils to avoid coil heating during measurements. Ten linearly spaced excitation frequencies between 10kHz and 200kHz were applied. A subset of the fabricated coils is shown in Figure 8. In total, nine realizations of the two-layer and eight-layer PCB coils and two realizations of the wire-wound coils were measured.

**Figure 9:**
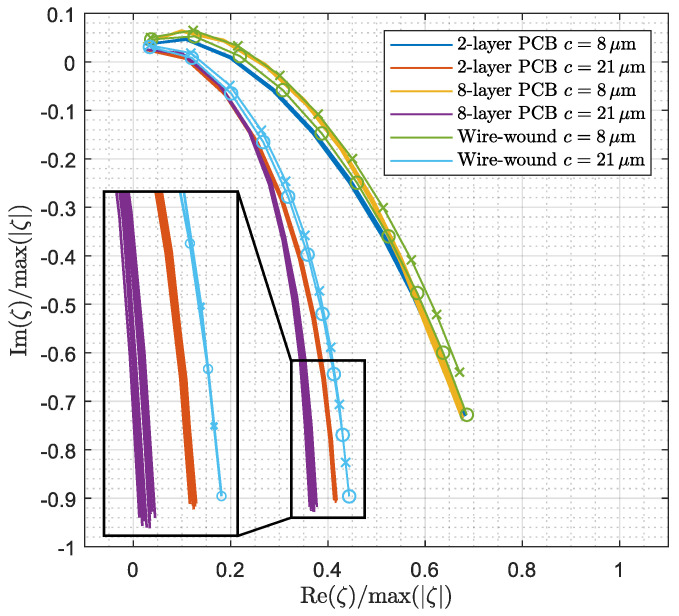
The families of curves of the normalized mutual impedance curves highlight the variability introduced by manufacturing tolerances across the coil configurations. Nine PCB coil realizations per design and two wire-wound realizations are examined.

Figure 9 shows the normalized mutual impedance in the complex plane for the two sheet samples and all coil realizations. The mutual impedance curves are normalized to the maximum magnitude |ζ|, resulting in $\zeta_{N}=\zeta /\max\left(|\zeta |\right).$ This yields a comparable scale across coils. The measurement noise is negligible due to the precise setup and robust signal processing. Geometry tolerances dominate the identified normalized variations. The two-layer PCB coils show the smallest deviation. The eight-layer PCB coils exhibit a larger scattering. Only two wire-wound coils were fabricated, so statistics are limited. For better visibility, these two curves are marked with x and o. Their deviations due to the manufacturing tolerances are large, exceeding those of the eight-layer PCB coils. This illustrates the strong scatter possible with wire-wound coils.

## 8. Reproducibility of Model-Based Coating Thickness Estimation

It is now of interest to examine how these scatterings in the mutual impedance plane affect the reproducibility of the model-based coating thickness estimation. To provide sufficient degrees of freedom, a least-squares problem in the form

(11) $$J_{\mathrm{meas}}=\sum_{f=f_{\min}}^{f_{\max}}{\left(\zeta_{\mathrm{model}}\left(f,c,L,\mu_{2}\right)-\zeta_{\mathrm{meas}}\left(f\right)\right)}^{2}$$

is used. By minimizing this cost function, estimates for the unknown parameters can be given by

(12) $$\left(\hat{c},\hat{L},{\hat{\mu }}_{2}\right)=\mathrm{argmin}\left(J_{\mathrm{meas}}\right).$$

Unlike the estimation problem in Section 4, the substrate permeability must also be estimated, since it is unknown, thereby providing the estimator with additional degrees of freedom. For magnetically linear substrate materials, knowledge of substrate properties is not required to obtain unbiased coating thickness estimates [27]. In this study, the steel substrate was assumed to exhibit approximately linear magnetic behavior. Therefore, the optimization problem must explicitly include this additional degree of freedom to ensure an unbiased coating thickness parameter estimation. Minimization was again performed by using a Levenberg–Marquardt algorithm [26]. Due to the high computational costs of the fine analytical PCB coil model, the uncertainty analysis for PCB coils was performed using the coarse analytical model to decrease computational costs while ensuring acceptable accuracy, as shown in Figure 5.

For model-based parameter estimation, the nominal coil geometry parameters were used, and no additional calibration was performed. The reproducibility was evaluated by analyzing the scattering of the estimated parameters obtained from different realizations of the same coil configuration with respect to their corresponding mean value.

Due to the limited number of fabricated wire-wound coils, the calculation of the standard deviation would not have been not meaningful. Therefore, an alternative evaluation criterion was employed. For the different samples and coil configurations, the maximum relative errors are summarized in Table 4 and defined as

(13) $$\epsilon_{\max}=\frac{\left|\max\left(\hat{c}\right)-\min\left(\hat{c}\right)\right|}{\bar{c}},$$

where $\bar{c}$ denotes the individual mean value of the respective thickness estimates.

**Figure 10:**
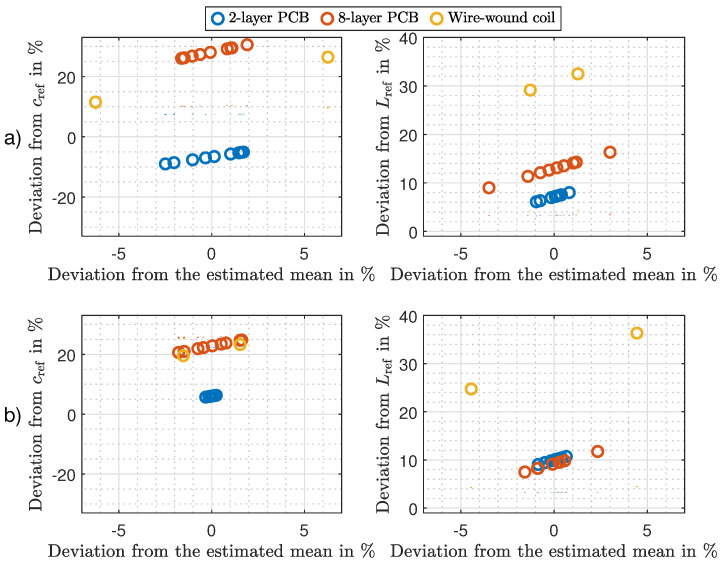
Scattering of the coating thickness and liftoff estimate for different sheet samples for the investigated manufactured coil setups: (**a**) c=8μm, (**b**) c=21μm.

In addition, the absolute values of the estimated parameters were considered and compared to the reference values of the coating thickness and the liftoff and are shown in the form of a relative deviation to the corresponding reference value. This allows an evaluation of the systematic bias of the measurement in comparison to the ground truth. The corresponding results for the two investigated samples are shown in Figure 10. The horizontal axis represents the relative deviation of the estimated parameters from their corresponding mean value in percent. The vertical axis shows the relative error of estimated parameter values to the reference values as individual points. Consequently, both the absolute deviation from the reference value and the spread around the corresponding mean value can be seen simultaneously.

It can be seen that the scattering from the estimated mean tends to increase for thinner coatings. Among all coil setups, the two-layer PCB achieves the best reproducibility on average and, therefore, the smallest maximum error $\epsilon_{\max}$. The eight-layer PCB shows a very similar maximum relative error for the 8μm coating and an increased error for the 21μm coating. For wire-wound coils, the reported error is likely underestimated, since only two coils were manufactured, and the normalization of the error to the mean $\bar{c}$ dampens the effect. Consistent with Figure 9, wire-wound coils show a much wider spread in the complex mutual-impedance plane. The smaller error observed with the 21μm coating can be attributed to the fact that the two underlying differential impedance curves are merely shifted on each other. The random shift caused by manufacturing inaccuracies has only a minor effect on the estimated coating thickness in this case, primarily influencing the liftoff estimation instead and showing a large scattering. With a larger sample size of wire-wound coils, a substantial rise in the error is expected. Given the way the error $\epsilon_{\max}$ is computed, it represents a lower bound and cannot be reduced by increasing the number of samples.

Furthermore, it can be observed that the estimated absolute values obtained with the two-layer PCB coil show very good agreement with the reference values for both the coating thickness and the liftoff. For the nominal liftoff of L=3mm, a slightly increased effective liftoff is expected in practice due to the surface roughness of the spacer, the sample surface, and the bottom side of the coil. This is also reflected in the estimated results of the two-layer and eight-layer PCB coils.

In contrast, the wire-wound coils and eight-layer PCB coils exhibit larger deviations in the absolute coating thickness estimates and generally tend to overestimate the parameters. These deviations are attributed to manufacturing inaccuracies and additional non-modeled effects, such as skin and proximity effects within the windings.

Consequently, the two-layer PCB coil clearly provides both the smallest scattering of the estimated parameters and the best agreement with the reference values without requiring additional calibration. Therefore, it shows the best manufacturing reproducibility and the lowest estimation bias among the investigated coil configurations.

An additional advantage of the two-layer PCB coil design is that, after fabrication, the total PCB thickness can be easily and non-destructively measured. This allows the actual overall PCB coil thickness $L_{2}$ to be directly incorporated into the model-based estimation approach. As a result, geometric uncertainties can be significantly reduced without the need for complex calibration procedures. Furthermore, due to the relatively small copper conductor thickness in the two-layer top and bottom configurations, variations in copper thickness only have a minor influence on the parameter estimation. In contrast, the accurate geometry calibration of an eight-layer PCB coil is considerably more challenging. Due to the large number of stacked layers, the exact internal structure cannot be reliably determined solely through external PCB thickness measurements. In practice, a destructive microsectioning analysis is required to obtain a precise representation of the layer stack-up. This significantly increases calibration effort and limits the practicality of accurately incorporating the real geometric configuration into a model-based estimation framework.

## 9. Conclusions

In this work, a detailed analytical eddy current mutual impedance model for differential multi-layer PCB pancake coils was derived, validated against finite element simulations, and benchmarked against the classical Dodd and Deeds coarse model approximation. Model studies indicate that the mutual impedance discrepancy between the fine and coarse models decreases with the number of PCB layers, while the applicability of the coarse model remains design- and stack-up-dependent and should be assessed case-by-case depending on the geometry and sample properties. The derived fine analytical PCB coil EC model can also be adapted to other ECT applications. For high-accuracy parameter estimation or modeling approaches, the presented PCB-coil model offers a powerful way to reduce modeling errors compared with the classical coarse-model approximations typically used. Measurements of metallic-coated steel sheets with different realizations of two- and eight-layer PCB coils and wire-wound coils demonstrate markedly reduced scattering in the complex mutual-impedance plane and consequently in the model-based coating thickness estimates when using PCB coils. The two-layer PCB achieves the best reproducibility and lowest estimation bias without calibration. For coating thicknesses of 8μm and 21μm, the maximum relative errors from the estimated mean, which are used as a lower bound, are $\epsilon_{\max}=4.48\%$ and 0.61% for two-layer PCBs, and 3.40% and 3.13% for eight-layer PCBs, while wire wound coils yield 12.31% and 3.07%. The latter are likely underestimated due to the small sample size and show a best-case error. Overall, lithographically defined PCB fabrication significantly improves reproducibility without compromising sensitivity to coating thickness. Consequently, for industrial deployments aiming for reproducible coating thickness estimates of thin metallic coatings, PCB coils should be preferred, as they obviate the need for laborious coil geometry calibration and deliver good reproducibility when the nominal coil geometry is used in a model-based estimation approach.

## Figures and Tables

**Table 1:** Used PCB stack-ups.

8-Layer PCB		
**Layer**	**Material**	**Thickness**
Top copper (L1)	Copper	35 µm
Dielectric	Prepreg (PR1080/2116)	0.19 mm
Inner copper (L2)	Copper	35 µm
Core	FR4-Improved	0.20 mm
Inner copper (L3)	Copper	35 µm
Dielectric	Prepreg (PR2116)	0.24 mm
Inner copper (L4)	Copper	35 µm
Core	FR4-Improved	0.20 mm
Inner copper (L5)	Copper	35 µm
Dielectric	Prepreg (PR2116)	0.24 mm
Inner copper (L6)	Copper	35 µm
Core	FR4-Improved	0.20 mm
Inner copper (L7)	Copper	35 µm
Dielectric	Prepreg (PR1080/2116)	0.19 mm
Bottom copper (L8)	Copper	35 µm
**2-layer PCB**		
Top copper (L1)	Copper	35 µm
Core	FR4-Improved	1.55 mm
Bottom copper (L2)	Copper	35 µm

**Table 2:** Geometry parameters and number of turns for the two PCB coil setups.

Layers	$N_{1}$	$N_{2}$	$r_{\mathrm{EC}}$	$r_{\mathrm{MC}}$	*s*	$t_{\mathrm{EC}}$	$t_{\mathrm{MC}}$
	-	-	mm	mm	mm	mm	mm
2	24	21	25.5	18	0.15	0.25	0.15
8	88	76	26.3	19.4	0.15	0.207	0.15

**Table 3:** Geometry parameters (mm) and number of turns for wire-wound coil setup.

Setup	$r_{1}$	$r_{2}$	$r_{3}$	$r_{4}$	$L_{2}$	$L_{5}$	$L_{6}$	$N_{1}$	$N_{2}$
wire-wound	24	24.9	23.7	24	10	0	5	11	17

**Table 4:** A comparison of the reproducibility and sensitivity of the investigated coil systems. Error values marked with * may be underestimated due to the small sample size. The standard deviation values marked with ** are based on a limited number of samples (n=2) and should be interpreted with caution.

	Wire-Wound		2-Layer PCB		8-Layer PCB	
$c_{\mathrm{ref}}$ in μm	8	21	8	21	8	21
$\epsilon_{\max}$ in %	12.31 *	3.07 *	4.48	0.61	3.40	3.13
σ in μm	0.843 **	0.552 **	0.118	0.039	0.128	0.316
|Sensitivity| in mV/μm	3.50		2.59		27.00	

## Data Availability

The data presented in this study are available on request from the corresponding author as they are subject to a pending patent application.
